# Editorial: Magnetic resonance and artificial intelligence: online guidance for adaptive radiotherapy in abdominal and pelvic cancer treatment

**DOI:** 10.3389/fonc.2024.1409109

**Published:** 2024-04-19

**Authors:** Marco Fusella, Tomas Janssen, Lorenzo Placidi

**Affiliations:** ^1^ Department of Radiation Oncology, Abano Terme Hospital, Abano Terme, Italy; ^2^ Department of Radiation Oncology, the Netherlands Cancer Institute, Amsterdam, Netherlands; ^3^ Department of Diagnostic Imaging, Oncological Radiotherapy and Hematology, Fondazione Policlinico Universitario Agostino Gemelli, IRCCS, Rome, Italy

**Keywords:** magnetic resonance imaging (MRI), artificial intelligence, online adaptive radiation therapy, pelvic cancer, abdominal cancer, radiotherapy

In recent years, we have seen the development and clinical introduction of integrated Magnetic Resonance Imaging (MRI) linear accelerators within the radiotherapy community. With MRI-guided radiotherapy (MRIgRT) high soft tissue contrast images can be acquired at several time points during the treatment. This enables daily online adaptation to anatomical changes between treatment fractions and monitoring of anatomical changes. By using delineations of the anatomy on images acquired just prior to the treatment, MRIgRT allows for the reduction of geometrical uncertainties due to interfraction motion. As a result, treatment margins can be reduced, allowing a reduction of radiation-induced toxicity. However, MRIgRT adaptive radiotherapy comes at a cost. Online delineation, treatment planning, and quality assurance are all time-consuming and operator-dependent steps, making treatment slots considerably longer and hampering staff and departmental efficiency. Automation, and specifically the use of machine learning and artificial intelligence, has shown great potential when it comes to streamlining these tasks, especially for online auto segmentation, synthetic CT generation (1), and intrafraction motion management (2). However, the impact of these tools in daily practice is still very much undeveloped, and they represent a great potential area for improvement in the management of common cancers.

The goal of this Research Topic is to collect and summarize the growing knowledge of using magnetic resonance and artificial intelligence for online guidance adaptive radiotherapy in abdominal and pelvic cancer treatment. Eight top-notch manuscripts are published in this Research Topic in Frontiers in Oncology – Radiation Oncology Journal.

In the first manuscript of this Research Topic, Zhou et al. developed a DL solution for automatic segmentation of abdominal organs at 0.35 T MR images. Multiple 2D UNet models were trained, and the best-performing one was identified and trained further using a 3D strategy. Evaluation metrics, including Dice Similarity Coefficient (DSC) and intersection over union (IOU), were used to assess the models. The reported results highlight that, even if the obtained segmentation for the duodenum were not optimal, the findings of the study imply a potential clinical application of the 3D nnUNet model for the segmentation of abdominal OARs for images from 0.35 T MR-Linac.

The second manuscript reports the experience of Lorenzen et al. The authors developed and assessed an automatic segmentation method using the nnUNet framework for 1.5 T MRI-Linac scans for patients with prostate, metastatic prostate, or bladder cancer. The network was trained on 38 patients and tested on 30 patients, demonstrating high accuracy with over 90% of contours showing a DSC above 0.9. The nnUnet test results outperforming the current clinical practice using DIR-based contour propagation at the 1.5 T MRI-linac. The authors state that the trained network is sufficiently fast and accurate for clinical use in an online setting for MRgRT.

In the third manuscript (the last one related to auto-contouring), Vagni et al. investigated the impact of bias field artifact correction on 0.35 T pelvis MRIs in 60 prostate cancer patients treated using their MRI-LINAC. This artifact arises from various sources, including sensitivity variations of the imaging system, magnetic field inhomogeneities, and patient-related factors leading to a gradual change in signal intensity, resulting in a non-uniform intensity distribution across the image that can obscure important structures, reduce contrast, and compromise the accuracy of image analysis techniques. Using the N4ITK algorithm, bias correction was applied, and a 3D GAN was trained to auto-segment the rectum and bladder. Evaluation metrics (DSC and HD95th) showed similar performance for both original and bias-corrected MRIs in the test set. The GAN was robust to the variations in the signal caused by the bias field artifact and therefore able to isolate the effect and to auto-segment the OARs with the same accuracy for both the corrected and not corrected MR volumes.

Safaa Tahri et al., in the fourth manuscript, reported the evaluation and assessment of a generic DL model for sCTs generation from various MRI devices in the context of MR-only radiotherapy for prostate cancer. The study involved 90 patients from three centers, with a 2D Pix2Pix GAN trained on 24 CT-MR prostate pairs for each of the seven supervised models. The accuracy of the obtained sCT, in terms of image and dose, is equivalent to whether MRI images are generated using the generic model or the monocentric model. The generic model offers robust sCT generation, facilitating prostate cancer MRI-only radiotherapy for routine clinical use.

In the fifth manuscript, Prunaretty et al. developed and clinically evaluated an AI-based sCT generated from low-field MR images for pelvic cancer care. Using TheraPanacea software and self-supervised GANs, an sCT was created from 350 pairs of weakly aligned planning CTs and 0.35T TrueFisp MRIs. Image accuracy was assessed with a retrospective cohort of 20 test cases from eight institutions and various CT vendors. Dosimetric evaluation involved 29 prostate cancer patients, with treatment plans optimized on planning CTs and recalculated on sCTs. The results of the study demonstrated the feasibility of generating clinically acceptable artificial intelligence-based pseudo-CTs for low MR in the pelvis with consistent image accuracy and dosimetric results.

The sixth manuscript reports the experience of Wu et al. and is a study conducted to predict the response of locally advanced rectal cancer (LARC) patients treated on a 1.5 T MR-Linac by assessing the feasibility of using delta radiomics as a predictive tool for pathological complete response (pCR) after neoadjuvant chemoradiotherapy (nCRT). T2-weighted images and corresponding targets were obtained from 28 patients at each fraction, and clinical variables were collected. Delta features, calculated from the ratio of each fraction’s radiomics features to the first fraction, were analyzed. The authors report that delta features based on MRI images acquired during a short-course MRgART could potentially be used to predict treatment response in LARC patients undergoing nCRT.

In the seventh manuscript, Rusu et al. investigated the dosimetric impact of intrafraction physiological organ changes during online adaptive radiation therapy for pancreatic cancer on an MR-Linac. The observed intrafraction motion can have a significant dosimetric impact; measures to mitigate this motion are therefore needed. The authors conclude that, despite consistent intrafraction motion, plan adaptation still provides a dosimetric benefit.

In the last manuscript, He et al. reviewed the current status and perspectives of research progress in prostate cancer using DL-based computer-aided diagnosis to make the AI process understandable to not only radiologists but also general physicians who lack systematic and specialized imaging interpretation training. In particular, the authors have briefly described the application of DL techniques based on prostate MRI data and provide possible research ideas for future studies. The authors conclude that with the advancements in technology and research, a significant leap in deep learning algorithm development will occur, which will be beneficial to prostate cancer diagnosis and treatment.

This editorial highlights some recent results in the field of magnetic resonance and AI for online adaptive radiotherapy in abdominal and pelvic cancer treatment. This Research Topic guides multidisciplinary teams, composed of physicians, medical physicists, therapists, and data scientists, who are developing, clinically implementing, and enhancing the use of MR and AI within the frame of online adaptive radiotherapy.

## Author contributions

MF: Conceptualization, Supervision, Writing – original draft, Writing – review & editing. TJ: Conceptualization, Supervision, Writing – original draft, Writing – review & editing. LP: Conceptualization, Supervision, Writing – original draft, Writing – review & editing.
